# Adjunctive Impact of Mitochondria-Targeting Antibiotics for Cancer Stem Cells of Oral Squamous Cell Carcinoma: Proposal for a Novel Approach in Resistant Cases

**DOI:** 10.30476/dentjods.2024.102852.2397

**Published:** 2024-09-01

**Authors:** Mohammad Amin Amiri, Hesam Abbasi, Shahram Hamedani, Hossein Daneste

**Affiliations:** 1 Oral and Dental Disease Research Center, School of Dentistry, Shiraz University of Medical Sciences, Shiraz, Iran; 2 Postgraduate Student, Dept. of Oral and Maxillofacial Surgery, School of Dentistry, Shiraz University of Medical Sciences, Shiraz, Iran; 3 Dept. of Oral and Maxillofacial Surgery, School of Dentistry, Shiraz University of Medical Sciences, Shiraz, Iran

## Dear Sir

Oral squamous cell carcinoma (OSCC) is the most common cancer involving the oral and maxillofacial area [ 1 ]. OSCC is reported to exert the worst quality of life among the patients suffering from head and neck cancers [ 2 ]. Moreover, the rate of survival in these cases and the prognosis of OSCC are the concerning issues among the oncologists and oral and maxillofacial surgeons [ 3 ]. The current proposed treatment options can be a combination of surgical removal, chemotherapy, and radiotherapy [ 4 - 5 ]. Despite the success of the mentioned treatment protocols employed in these cases, many patients still suffer from low quality of life and short-time survival rate after the surgeries in resistant and metastatic cases [ 2 , 6 - 7 ]. These shortcomings have motivated scientists to look for novel treatment options to alleviate the symptoms and improve the patients’ quality of life, especially in resistant and recurrent cases [ 7 ].

One of the noticeable parts of OSCC leading to its development, metastasis, chemo-resistance, and survival is the cancer stem cells (CSCs) at the core of the tumor [ 8 - 9 ]. CSCs are highly potent cells resembling other stem cells in terms of self-renewal, producing heterogeneous and differentiated cells [ 10 ]. These CSCs have been shown to have an exceptionally high potential for survival and proliferation in hypoxic conditions and under high doses of medications [ 11 ]. The study of these CSCs can provide valuable insights into the prognosis and chemoresistance potential of OSCC [ 12 ]. 

Due to the high potency of CSCs and their critical role in the development of cancers, especially OSCC, few treatment options are suggested to minimize the activity and survival of CSCs [ 10 , 13 ]. In this regard, targeting the CSCs surface markers, signaling pathways, miRNA-based treatments, and immunotherapy are the proposed strategies to deal with CSCs [ 10 ]. Among the mentioned treatment options, the application of antibiotics can be a possible option due to their advantage of availability, low-cost, and their extensively studied mechanisms of action [ 14 ]. Concurrently, the antibiotics that target the mitochondria are shown to be a viable option. In the study by Lamb *et al*. [ 14 ], it was demonstrated that erythromycins, tetracyclines, glycylcyclines, an antifungal drug, and chloramphenicol are the groups of antibiotics, which have been proven to be effective in this regard. Among these groups, azithromycin, doxycycline, tigecycline, pyrvinium pamoate, and chloramphenicol were verified to be the proof-of-concept examples [ 14 ]. Concerning the mechanism of action, it is demonstrated that erythromycin and chloramphenicol target 39S large mitochondrial ribosome, tetracycline and glycylcyclines target 28S small mitochondrial ribosome, and pyrvinium pamoate targets the mitochondrial oxidative phosphorylation system [ 14 ]. In general, the application of these types of antibiotics results in inhibition of mitochondrial biogenesis or oxidative phosphorylation system [ 14 ]. 

The emergence of resistant cases is due to the genetic variations of the cancer genotype to optimally adjust to the new environment [ 15 ]. Therefore, the approach of phenotypic targeting (instead of genotypic) of cancer stem cells would be a novel solution in resistant cases with OSCC. The logic behind this approach arises from the fact that all the genetic changes making the tumor resistant against various treatments are ascribed to the presence of CSCs [ 14 ]. Therefore, targeting the CSCs would probably provide a novel treatment option for OSCC. Furthermore, this concept can be likely employed, as a preventive approach in avoiding the possible metastasis or recurrence in OSCC.

Application of mitochondria-targeting antibiotics in patients suffering from OSCC may provide a possible innovative option in minimizing the activity of CSCs in OSCC tumors, which would finally result in prevention of further metastasis, tumor recurrence, genetic-based tumor resistance, and tumor development. Further studies in *in vitro* and *in vivo* settings are encouraged to evaluate the prospect of this hypothesis.
